# Cofilin 2 Acts as an Inflammatory Linker Between Chronic Periodontitis and Alzheimer’s Disease in Amyloid Precursor Protein/Presenilin 1 Mice

**DOI:** 10.3389/fnmol.2021.728184

**Published:** 2021-09-30

**Authors:** Qing Zeng, Qin Fang, Xincai Zhou, Hongfa Yang, Yang Dou, Wenhao Zhang, Pu Gong, Xianfang Rong

**Affiliations:** ^1^Department of Stomatology, Shenzhen Baoan Women’s and Children’s Hospital, Jinan University, Shenzhen, China; ^2^Department of Cardiology, The Second Affiliated Hospital of University of South China, Hengyang, China

**Keywords:** chronic periodontitis, Alzheimer’s disease, 2D-DIGE, Cofilin 2, neuroinflammation

## Abstract

Increasing evidence has shown a correlation between chronic periodontitis (CP) and Alzheimer’s disease (AD). Nevertheless, there is still a lack of direct evidence, and especially key molecules to connect the two diseases. This study aims to investigate potential protein links between CP and AD within the inflammatory aspect. The hippocampus of CP model mice and controls were collected, and changes in protein expression were evaluated using two-dimensional differential in-gel electrophoresis (2D-DIGE) analysis combined with liquid chromatography tandem mass spectrometry. A total of 15 differentially expressed proteins were identified in CP model mice, as compared with the controls. Among them, S100-A9, transthyretin, Cofilin 2, peroxiredoxin 2, and lipocalin-2 were validated by Western blot according to their dual function both in inflammation and AD. Based on 2D-DIGE analysis, CP animal model had higher levels of S100-A9, Cofilin 2, peroxiredoxin 2, and lipocalin-2 compared to controls. The level of Cofilin 2, one of the well-established proteins in the pathology of AD, was strongly correlated with the time course of CP pathology, indicating a specific molecular correlation between CP and AD. Moreover, the *in vivo* results showed the level of Cofilin 2 increased significantly along with a prominent increase of the phosphorylation of protein phosphatase 2 (PP2A) and tau protein in the cell lysates of Porphyromonas gingivalis (P.g-LPS)-treated SK-N-SH APPwt cells. Cofilin 2 inhibition resulted in a sharp decrease in PP2A dependent of tau phosphorylation. Furthermore, tumor growth factor (TGF)-β1 was one of the most important inflammatory cytokines for the Pg-LPS-induced Cofilin 2 upregulation in SK-N-SH APPwt cells. These results showed inflammation served as the bond between CP and AD, whereas inflammatory related proteins could be the key linkers between the two diseases. Determining the association between CP and AD at the molecular mechanism will not only hold the direct evidence of the association between the two diseases but also provide a new way of preventing and treating AD: the effective prevention and treatment of CP could serve as a useful method to alleviate the development of AD.

## Introduction

Alzheimer’s disease (AD) is the most common neurodegenerative disease that seriously affects the physical and mental health of the elderly and results in heavy burden to families and society (Pudelewicz et al., 2019). At present, there is no definite conclusion about the etiology and pathogenesis of AD. Several theories have been postulated according to the genetic characteristics, clinical manifestations, pathological characteristics and other risk factors of AD, including cholinergic theory, amyloid cascade hypothesis, genetic theory, and apoptosis theory (Hardy and Higgins, 1992; Calsolaro and Edison, 2016; Armstrong, 2019). These events will eventually lead to synapse loss, neuronal apoptosis, brain volume reduction and finally dementia. However, no hypothesis can fully explain the etiology of AD (Gong et al., 2018). Considering the complications of AD pathology, apart from the pathogenic factors that are already known, other factors also exist (Cubinkova et al., 2018). Accordingly, the discovery of novel AD-related pathogenic factors and proteins will provide new insight into the molecular changes in the pathology of AD, which is critical for determining the patho-physiology of this disease (Arshavsky, 2020; Matsushita et al., 2020).

AD inflammation hypothesis is one of the three major hypotheses of AD pathogenesis. This hypothesis holds the notion that when the brain is infected, immune cells (mainly microglia) are activated to clear the source of infection. However, if the immune cells are over-activated, the brain will produce and release inflammatory response, which will attack the most vulnerable axons, eventually leading to the loss of prominence and neuronal cell death (Regen et al., 2017; Sochocka et al., 2017). Oral infection, especially chronic periodontitis (CP), is one of the main sources of neuro-inflammation in the brain (Nyariki et al., 2019). Periodontitis is a chronic infectious disease of periodontal tissue, which often leads to the destruction of periodontal support tissue and tooth loss. It is characterized by the increase of C-reactive protein (CRP) level and the release of pro-inflammatory cytokines into the systemic circulation (Esteves-Lima et al., 2020; Kozak et al., 2020).

Numerous studies have shown that CP has a strong correlation with AD: AD patients have a higher tendency of acquiring CP than non-AD patients of the same age; alternatively, CP patients have a higher risk of AD than healthy controls of the same age (Teixeira et al., 2017; Liccardo et al., 2020). Inflammation and bacterial infection are associated with CP and AD. On the one hand, pro-inflammatory factors produced by CP, such as interleukin (IL)-1 β, IL-6, IL-8, tumor necrosis factor (TNF)-α, and CRP, can reach the central nervous system (CNS) through systemic circulation, breaking the balance of inflammation in the brain (Rokad et al., 2017; Qiu et al., 2021). These inflammatory factors activate neuralgia through synergistic action and cause a cascade reaction that is conducive to the development of AD (Kantarci et al., 2020). On the other hand, periodontal bacteria or their toxic components (such as lipopolysaccharide) can also penetrate into the CNS through blood flow or peripheral nerve, induce microglia to release inducible nitric oxide syntheses and prostaglandin E2, destroy synaptic transmission of neurons, and cause cognitive impairment (Dioguardi et al., 2020). However, the current study in this area is descriptive, and there is no report on the key molecules and signaling pathways between the two diseases.

In the present study, we design our experiment with the following three aims: (i) investigate the association between CP and AD using an animal model, (ii) identify and validate differentially expressed proteins in CP animal model, and (iii) explore the molecular mechanism of Cofilin 2 as a protein link between CP and AD *in vitro*.

## Materials and Methods

### Animals

Amyloid Precursor Protein/Presenilin 1 (APP/PS1) mice (APP KM670/671NL, PSEN1deltaE9) and the wild-type littermate were purchased from The Jackson Laboratory. The animals were housed according to the standard mice housing procedures. The process of CP mice model construction has been described in previous report (Qian et al., 2021). Briefly, P.g-LPS (2 μl, 1 mg/ml) was injected into the palatal side of bilateral maxillary first and second molars of 3 month old APP/PS1 mice (3 times/week) and 4–0 silk thread was ligated on bilateral maxillary second molars for 3 month. All experiments were approved by Medical ethics committee of Shenzhen Baoan Women’s and Children’s Hospital.

### Behavioral Experiment

The Morris water maze task was used to evaluate the CP-related cognitive impairment of mice and the process was described in previous paper (Peng et al., 2012). Briefly, APP/PS1 mice were gently released into the water from one of the four positions and the swimming paths were monitored by a video camera on the top of the water maze. The training was performed for 5 consecutive days. Each day, training consisted of 2 blocks with each inter-block interval being 6 h. A maximum of 60 s was given to each mouse to find the hidden platform. If failed, the training was terminated and 60 s was assigned, and the mouse was manually guided to the hidden platform. The mouse was allowed to stay on the platform for 10 s before it was sent back to the cages.

### Two-Dimensional Differential In-Gel Electrophoresis and LC-MS/MS Analysis

Two-dimensional differential in-gel electrophoresis (2D-DIGE) and liquid chromatography-tandem mass spectrometry (LC-MS/MS) analysis and LC-MS/MS were performed according to the protocol described previously (Rong et al., 2020). Typically, to increase the signal consistency of each gel, the photomultiplier tube (PMT) was set to ensure maximum pixel intensity values for all gel images within a range of 40,000–60,000 pixels. DeCyder 7.0 (GE Healthcare) was used to analyze the images. In differential in-gel analysis (DIA) module, spots were detected, matched and normalized; in biological variation analysis (BVA) module, spot statistics were reviewed. The spots with average ratio more than +2 or less than −2 and with statistical difference (*p*< 0.05) were isolated for identification. LC-MS/MS analysis was carried out using a Surveyor MS Pump Plus HPLC system coupled to a Thermo Fisher Finnigan LTQ linear ion trap mass spectrometer (Thermo Fisher Corporation, San Jose, CA) using nano-electrospray ionization.

### Western Blot Analysis

The brain of the APP/PS1 mice were removed and dissected for hippocampus on ice, and then homogenized thoroughly in the RIPA lysis buffer [150 mMNaCl, 50 mMTris (pH 7.4), 1% NP40, 0.5% sodium deoxycholate, and 0.1% SDS]. All the samples were subjected to electrophoresis, transferred onto PVDF membranes and incubated with anti-S100A9 (CST, 73425), anti-transthyretin (Santa Cruz, sc-377517), anti-Cofilin 2 (Santa Cruz, sc-166958), and anti-peroxiredoxin 2 (CST, 46855), Anti-lipocalin-2 (abcam, ab216462), and anti-β-actin (CST, 3700) overnight at 4°C. After washing with TBST for 5 times, HRP-coupled secondary antibodies (CST, 7074 or 7076) were applied at room temperature for 2 h with gentle agitation. The signal was detected with LAS4000 Fuji Film imaging system (Fuji Film, Japan) and analyzed with Image J software.

### Cell Culture

Human neuroblastoma SK-N-SH cells overexpressing wild-type APP695 (SK-N-SH APPwt) were grown as previously report (Peng et al., 2014). For CP cell model, SK-N-SH APPwt cells were treated with 1 μg/ml P.g-LPS (Invivo Gen, San Diego, United States) for 7 days (subculture once). For Cofilin 2 activation, IL-6 (10 ng/ml, Genscript, Z03134), IL-1β (100 pg/ml, Genscript, Z02978), recombinant Human CRP protein (1 mg/L, Abcam ab171471), TNF-α (10 ng/ml, Genscript, Z01001), and TGF-β1 (10 ng/ml, Genscript, Z03441) were applied to SK-N-SH APPwt cells for 24 h. For TGF-1β inhibition, SB-505124 (10 μM, Selleck, S2186) was applied to P.g-LPS treated SK-N-SH APPwt cells 24 h before cell harvest.

### Immunostaining Analysis

At the confluence of 30–50%, the medium of SK-N-SH APPwt cells was removed. The cells were washed three times with PBS and fixed in 4% paraformaldehyde for 30 min. After fixation, cells were thoroughly rinsed with PBS and incubated with goat serum in PBS containing 0.5% Triton X-100 for 2 h at room temperature. Then, the cells were incubated with an AT8 antibody (Thermo Fisher Scientific, 1:200) overnight at 4°C. The cells were rinsed with PBS for three times, followed by incubating with secondary antibodies [Fluorescent-labeling AlexaFluor 488 (anti-mouse, 1:200)] (2 h, RT). The immuno-staining images were performed by fluorescence microscope (Nikon Eclipse Ti-S) after treatment with PI for 5 min to label nuclear DNA.

### RNA Interference

For Cofilin 2 knockdown, specific siRNAs for human Cofilin 2 (Santa Cruz, sc-37027), and control (Santa Cruz, sc-37007) were mixed with lipofectamine RNAiMAX (Invitrogen) under the previous transfection protocol according to the manufacturer’s specification. The mixture was incubated in a final volume of 1 ml for 20 min and then added to around 80% confluent SK-N-SH APPwt cells in six-well plates for a final volume of 3 ml. Five hours later, the medium was changed to the fresh complete medium for an additional 43 h before cells were harvested.

### Co-immunoprecipitation

Co-immunoprecipitation was carried out using protein A/G PLUS-agarose immunoprecipitation reagent (sc-2003, Santa Cruz) according to the manufacture’s instruction. Briefly, the lysis were pre-cleared with 20 μl protein A/G PLUS-agarose, together with 1 μg species-matched control IgG for 30 min at 4°C. To detect the proteins that could interact with cofilin 2 or PP2A, the lysis (around 500 μg) were incubated with 2 μg anti-Cofilin 2 or anti-PP2A antibody for 2 h with rocking at 4°C. Then, 20 μl protein A/G PLUS-agarose was added and incubated overnight with rocking at 4°C. The imunnoprecipitates were pelleted and washed five times by RIPA buffer. The precipitates were resolved by 40 μl of 1×electrophoresis sample buffer and then subjected to Western blotting analysis.

### Statistical Analysis

For the 2D-DIGE experiment, DeCyder 7.0 (GE Healthcare) was used to analysis data from DIGE (DIA and BVA model). For Western blot data, One-way ANOVA with SPSS version 13.0 (SPSS Inc., Chicago, IL, United States) was used for the analysis of the data. The differences between the groups were analyzed by Bonferroni’s *post hoc* test. All data were shown as mean ± SD and the control group was normalized to 100%. Prism software (GraphPad Prism 5, La Jolla, CA, United States) was used to create the pictures. A value of *p*< 0.05 was considered to be statistically significant.

## Results

### Chronic Periodontitis Deteriorated the Spatial Learning and Memory Deficits in Amyloid Precursor Protein/Presenilin 1 Mice

**FIGURE 1:**
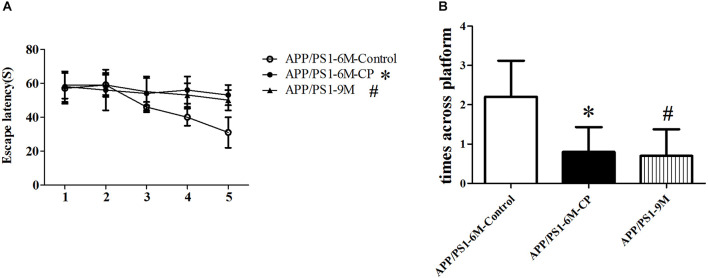
CP deteriorated the spatial learning and memory deficits in APP/PS1 mice. The spatial learning and memory was assessed by the Morris water maze task. **(A)** Latency score represents the time taken to the platform once the mouse was put in the water. **(B)** Times across the platform represent the frequency for a mouse to swim across the circle where the hidden platform located previously. CP significantly deteriorated attenuated the learning and memory deficits as compared to control mice. Data represent mean ± SD. *n* =16 mice per group. ^∗^*p* < 0.05 vs. control group. ^#^*p* < 0.05 vs. control group.

Spatial learning was assessed by the Morris water maze with the time required to determine the hidden platform and the times across the circle where the hidden platform was located previously. Repeated measures analysis of variance revealed a significant day effect on escape latency within the two groups: CP mice had evident cognitive impairment at the age of 6 months, requiring a significant amount of time to discover the platform (*p*< 0.05), suggesting CP could worsen the spatial learning effectively across the 5-day training period (Figure 1A). Furthermore, probe trials were conducted to assess the memory retention at 2 h after the last training session. The average crossing time was only 0.7 times for CP mice, whereas the control mice can cross the invisible platform 2.1 times in 60 s (*p*< 0.05, Figure 1B). For the two experiments above, 9-months old APP/PS1 mice were used as the positive controls, which showed a prominent cognitive impairment as compared to 6-month old APP/PS1 mice (*p*< 0.05, Figure 1).

### Identification of Differentially Expressed Proteins in Chronic Periodontitis Animal Model

**FIGURE 2:**
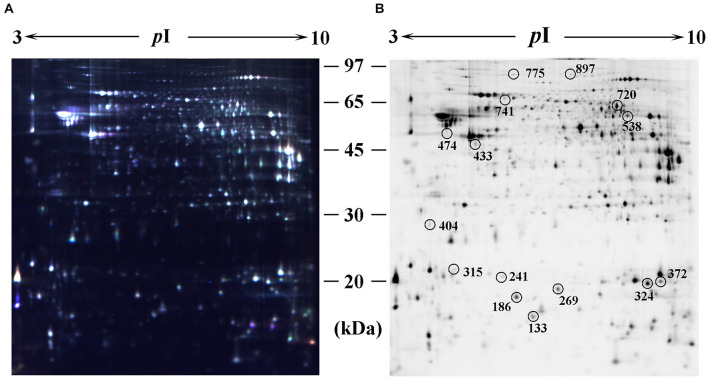
Identification of differentially expressed spots by using 2D-DIGE. Abundant hippocampus proteins were subjected to 2D-DIGE quantitative analysis to identify proteins with differential expression between CP group and control group. **(A)** Over-lay of cy2-marked loading control, cy3-marked and cy5-marked sample from CP mice or controls (*n* = 12). **(B)** Distribution of differentially expressed protein spots. The samples were separated using IPG gel (pH 3–10, 18 cm) in the first phase and 12.5% SDS-PAGE; 150 ug of protein was used in each gel. The spots showing significant differences between CP mice or controls (see Table 1) were labeled in a 2D-DIGE gel.

Proteins from the hippocampus of 12 CP mice and 12 control mice were analyzed using two-dimensional differential in-gel electrophoresis (2D-DIGE). For each gel, three images were generated to the three samples (CP mice, control mice, and internal standard). A representative DIGE gel showing the overlay of Cy3, Cy5, and Cy2 images from one such gel is shown in Figure 2A. In DIA workspaces, approximately 1,600 spots were detected in each gel using DeCyder software. In BVA module, Cy2 image from gel number 5 was chosen as a master gel as it had the maximum number of spots. Fifteen spots were found to be differentially expressed with the criteria. Among them, ten protein spots (spots 133, 241, 269, 315, 372, 433, 474, 538, 720, and 741) were up-regulated, and five pots (186, 324, 404, 775, and 897) were down-regulated. The differentially expressed spots in the hippocampus of CP mice and control mice were marked with black circles in pick gel (Figure 2B). The 15 spots of interest were manually excised from colloidal coomassie stained preparative gels of pooled samples for in-gel trypsin proteolysis and subsequently liquid chromatography tandem mass spectrometry (linear trap quadrupole) analysis. These 15 differentially expressed protein spots corresponded to 15 different proteins. All of these proteins were consistent with theoretical molecular weights and *pI* ranges based on the positions of spots on the gel (Table 1).

**TABLE 1:** Differentially expressed proteins in the hippocampus of CP mice model as compared with controls.

Spot no.^*a*^	Protein definition	Accession GI no.	Score	Sequence coverage (%)	p*I*^*b*^	MW^*c*^	Ratio	*p*-value
133	S100-A9	529367290	30.62	21.4	6.65	13048.9	+9.65	0.0084
186	Profilin 2	239937460	62.34	37.8	6.55	15032.2	−2.55	0.0472
241	Transthyretin	291463252	26.17	15.6	5.77	15775.9	+2.30	0.0019
269	Cofilin 2	126513132	35.20	30.4	7.66	18709.6	+14.2	0.0027
315	Peroxiredoxin 2	959092745	126.35	67.2	5.20	21778.7	+8.84	0.0024
324	Glutathione peroxidase 4	1547242141	31.69	25.7	8.78	22228.7	−2.26	0.0110
372	Lipocalin-2	124298099	102.58	50.3	8.96	22875.0	+10,000	0.0004
404	14-3-3 protein sigma	134023661	100.68	64.1	4.72	27706.0	−23.61	0.0383
433	Fibrinogen gamma chain	952977826	42.77	29.6	5.54	49391.4	+2.16	0.0237
474	Alpha-tubulin 2	111186463	23.64	15.2	4.98	49959.6	+4.98	0.0061
538	Synapsin-2 isoform IIb	557357673	50.36	33.5	8.59	63372.6	+4.85	0.0054
720	Keratin, type II cytoskeletal	126116584	110.50	56.7	8.39	65605.9	+2.64	0.0135
741	Prothrombin	805299479	33.61	20.8	6.04	70268.7	+3.60	0.0019
775	Glycogen phosphorylase	24,418,918	35.24	26.5	6.28	96730.0	−6.77	0.0036
897	Dynamin-1	684179330	72.50	43.4	7.61	97802.8	−3.28	0.0058

*Proteins from the hippocampus of CP mice model and controls were separated by DIGE. Their identities were determined by LC-MS/MS as described in section “Materials and Methods.”*

*^*a*^Spot numbers correspond to those in Figure 2B.*

*^*b*^pI is the theoretical calculated from the amino acid sequence of the predicted mature protein.*

*^*c*^MW refers to the theoretical molecular mass is kDa calculated from the amino acid sequence.*

### Western Blot Analysis for Validation

**FIGURE 3:**
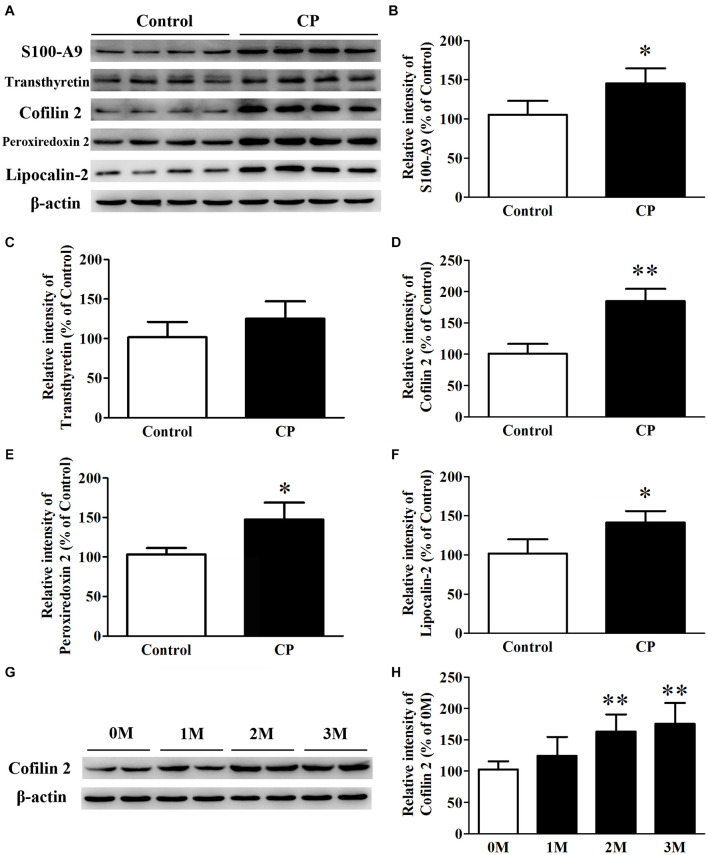
Expression of S100-A9, transthyretin, Cofilin 2, peroxiredoxin 2 and lipocalin-2 in the hippocampus of CP, and control mice. **(A)** Representative panel of Western blots of S100-A9, transthyretin, Cofilin 2, peroxiredoxin 2, and lipocalin-2. **(B)** Quantitative comparison of the Western blot of S100-A9. **(C)** Quantitative comparison of the Western blot of transthyretin. **(D)** Quantitative comparison of the Western blot of Cofilin 2. **(E)** Quantitative comparison of the Western blot of peroxiredoxin 2. **(F)** Quantitative comparison of the Western blot of lipocalin-2. **(G)** Representative panel of Western blots of Cofilin 2 in the time course of CP. **(H)** Quantitative comparison of the Western blot of Cofilin 2 in the time course of CP. Data represent mean ± SD for 8 mice per group. ^∗^*p* < 0.05 vs. control group; ^∗∗^*p*< 0.01 compared with 0M.

Five differential proteins were selected for validation by western blot analysis because of their well-established dual function both in inflammation and AD. As shown in Figure 3, consistent with the 2D-DIGE study, compared with the control mice, the expression of S100-A9, Cofilin 2, peroxiredoxin 2, and lipocalin-2 in the hippocampus of CP mice increased by 42.3% (*p*< 0.05), 85.1% (*p*< 0.01), 40.6% (*p*< 0.05), and 37.8% (*p*< 0.05), respectively (Figures 3B,D–F). Nevertheless, no significant change was observed for transthyretin level between the two groups (Figure 3C). Cofilin2 was selected for further validation. The results showed that Cofilin 2 level continued to rise along with the time course of CP pathology within the animal model (*p*< 0.05), suggesting a specific role for Cofilin 2 in the pathology of CP (Figures 3G,H).

### Cofilin2 Facilitated the Hyperphosphorylation of Tau Through Protein Phosphatase 2

**FIGURE 4:**
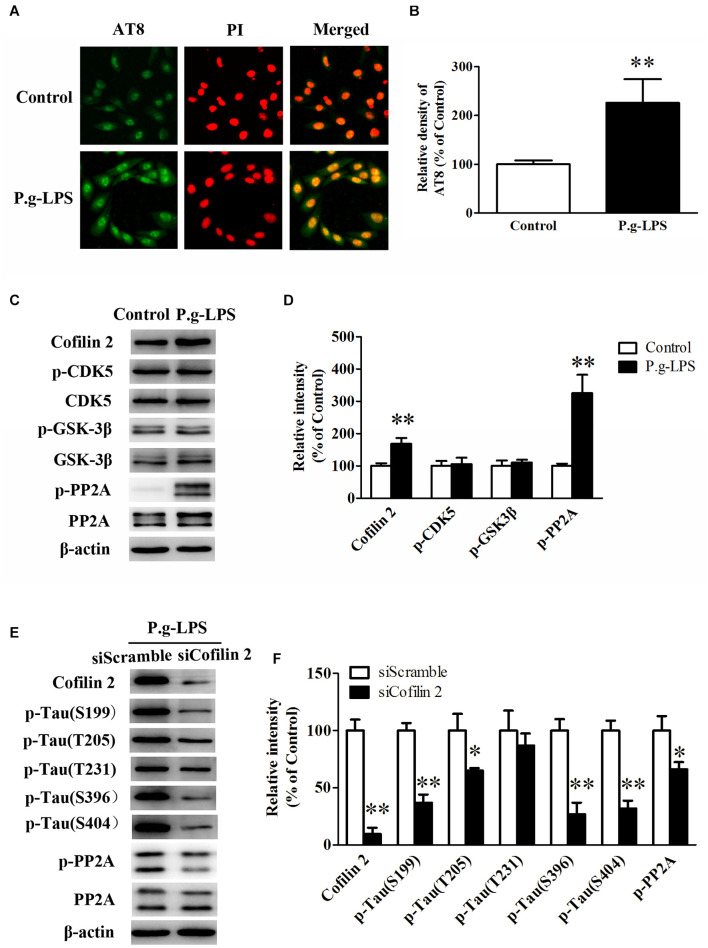
Cofilin 2 facilitated the hyperphosphorylation of tau through PP2A. **(A)** Immunofluorescence staining of AT8 in control and P.g-LPS treated SK-N-SH APPwt cells. **(B)** Quantitative comparison of the immunofluorescence staining of AT8 in control and P.g-LPS treated SK-N-SH APPwt cells. **(C)** Representative panel of the Western blot of Cofilin 2, p-CDK5, CDK5, p-GSK-3β, GSK-3β, p-PP2A, and PP2A in control and P.g-LPS treated SK-N-SH APPwt cells. **(D)** Quantitative comparison of the Western blot of Cofilin 2, p-CDK5, CDK5, p-GSK-3β, GSK-3β, p-PP2A, and PP2A in control and P.g-LPS treated SK-N-SH APPwt cells. **(E)** Representative panel of the Western blot of Cofilin 2, tau phosphorylation at Ser199, Thr205, Thr231, Ser396, and Ser404, p-PP2A, and PP2A in siScramble group and siCofilin 2 group. **(F)** Quantitative comparison of the Western blot of Cofilin 2, tau phosphorylation at Ser199, Thr205, Thr231, Ser396, and Ser404, p-PP2A, and PP2A in siScramble group and siCofilin 2 group. Data represent mean ± SD. ^∗^*p*< 0.05 vs. control group, ^∗∗^*p*< 0.01 vs. control group.

SK-N-SH APPwt cells were treated with 1 μg/ml lipopolysaccharide from Porphyromonas gingivalis (P.g-LPS) for 72 h to establish a CP cell model. The immuno-fluorescence results showed that Pg-LPS treatment significantly increased AT8 staining (ser202/thr205) in SK-N-SH APPwt cells (*p*< 0.01, Figures 4A,B). Subsequently, we examined the effect of P.g-LPS on the activity of glycogen synthase kinase 3 beta (GSK-3β), cyclin-dependent kinase 5 (CDK-5), and protein phosphatase 2 (PP2A), three major kinases for phosphorylation or de-phosphorylation of tau at abundant epitopes. The results showed the level of phosphor-PP2A (inactive form) was obviously evidently higher in the Pg-LPS group compared to the control group (*p*< 0.01). Conversely, phosphor-GSK-3β (Ser9) level and phosphor-CDK-5 expression remained relatively unchanged between the two groups (Figures 4C,D).

In addition, siCofilin 2 was applied in the supernatant of CP cell model. Quantitative analysis showed that the levels of tau phosphorylation at Ser199, Thr205, Thr231, Ser396, and Ser404 were lower by 67.3% (*p*< 0.01), 36.5% (*p*< 0.05), 12.6%, 74.2% (*p*< 0.01), and 70.3% (*p*< 0.01), respectively, compared to the siControl group (Figures 4E,F). Furthermore, we identified the molecular mechanism of sicofilin 2-mediated tau de-phosphorylation. The results showed that the level of phosphor-PP2A (inactive form) was evidently lower in the siCofilin 2 group compared to the siScramble group (*p*< 0.05) (Figures 4E,F). In contrast, there was no significant difference in phosphor-GSK-3β (Ser9) and phosphor-CDK-5 between the two groups (data not shown).

### Cofilin 2 Co-localized and Interacted With Protein Phosphatase 2

**FIGURE 5:**
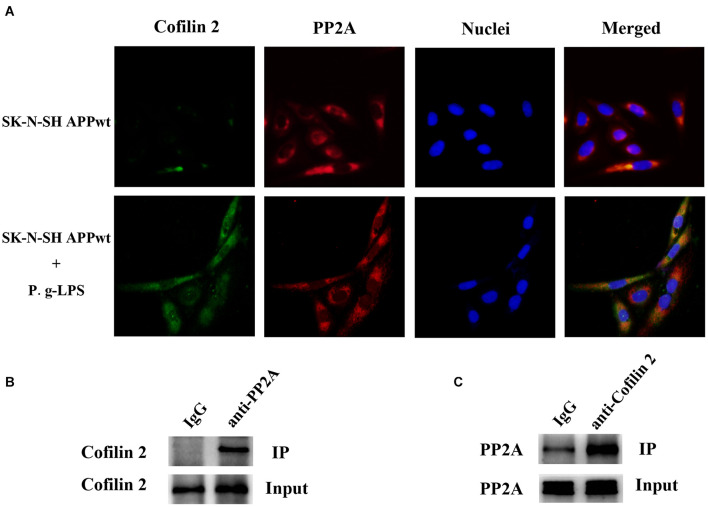
Cofilin 2 co-localized and interacted with PP2A. **(A)** Interaction between Cofilin 2 and PP2A was determined by co-immunofluorescence staining (co-IF) in SK-N-SH APPwt cells and P.g-LPS treated SK-N-SH APPwt cells. **(B)** Representative panel of the Western blot of cofilin 2 in IgG group and anti-PP2A group after co-immunoprecipitation (co-IP) in P.g-LPS treated SK-N-SH APPwt cells. **(C)** Representative panel of the Western blot of PP2A in IgG group and anti-cofilin 2 group after co-immunoprecipitation (co-IP) in P.g-LPS treated SK-N-SH APPwt cells.

Previous studies on the function of Cofilin 2 have demonstrated that this protein might interact with other molecules and exert its biological function (Sudnitsyna et al., 2012; Hayakawa et al., 2014). Therefore, we aimed to determine whether Cofilin 2 interacted with PP2A. Immuno-fluorescence images demonstrated a high level of co-localization between Cofilin 2 and PP2A in Pg-LPS-treated SK-N-SH APPwt cells, while in the control group, the overlay of the fluorescence from Cofilin 2 and PP2A could be barely detected (Figure 5A). Co-IP was used to verify the complex formation of the two proteins, and we found that Cofilin 2 certainly interacted with PP2A in P.g-LPS treated SK-N-SH APPwt cells with high affinity (Figure 5B). Moreover, to further support the finding, anti-Cofilin 2 antibody was applied to Co-IP experiment, and a highly significant binding between Cofilin 2 and PP2A could be observed equally (Figure 5C). A possible explanation could be that Cofilin2 interacted with PP2A and consequently inhibited the activity of PP2A. However, the details of this mechanism remain to be studied.

### Tumor Growth Factor-β1 Was Involved in Cofilin 2 Upregulation in Porphyromonas Gingivalis Lipopolysaccharide Treated SK-N-SH APPwt Cells

**FIGURE 6:**
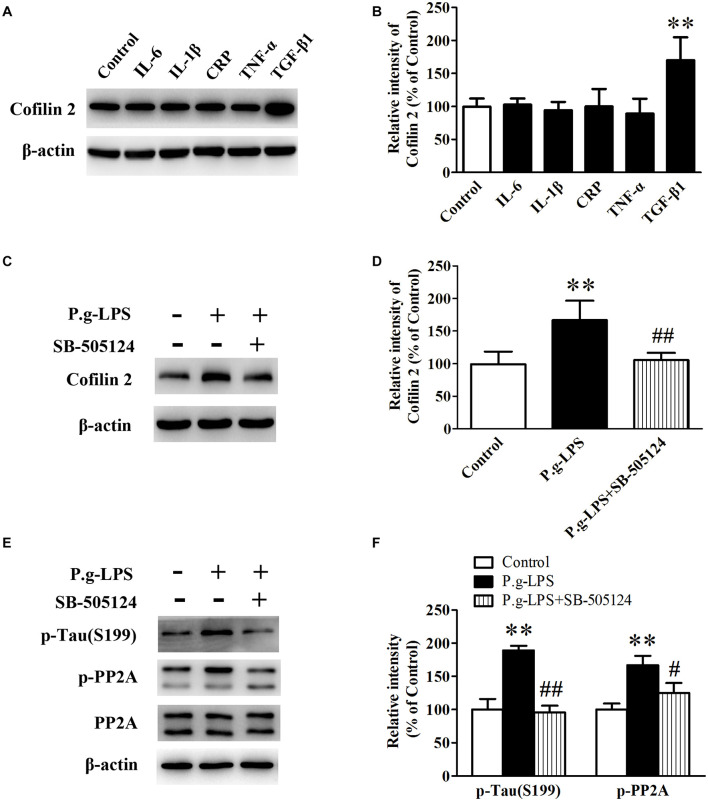
TGF-β1 was involved in Cofilin 2 upregulation in P.g-LPS treated SK-N-SH APPwt cells. **(A,B)** Representative panel and quantitative comparison of the Western blot of Cofilin 2 in IL-6-treated group, IL-1β-treated group, recombinant Human CRP-treated group, TNF-a-treated group, TGF-β1-treated group and Control group. **(C,D)** Representative panel and quantitative comparison of the Western blot of Cofilin 2 in Control group, P.g-LPS-treated group, and P.g-LPS plus SB-505124-treated group. **(E,F)** Representative panel and quantitative comparison of the Western blot of p-Tau (Ser199) and p-PP2A in Control group, P.g-LPS-treated group, and P.g-LPS plus SB-505124-treated group Data represent mean ± SD. ^∗∗^*p*< 0.01 compared with control group, ^#^*p*< 0.05 compared to P.g-LPS group, ^##^*p*< 0.01 compared to P.g-LPS group.

To elucidate the underlying mechanism of Pg-LPS-induced Cofilin 2 upregulation in SK-N-SH APPwt cells, the well-known inflammatory cytokines in CP pathogenesis including IL-6, IL-1β, CRP, TNF-α, and TGF-β1 were applied to the supernatant of SK-N-SH APPwt cells. Only TGF-1β treatment could significantly increase the protein level of Cofilin 2 (*p*< 0.01, Figures 6A,B). Moreover, SB-505124, a specific TGF-β1 receptor inhibitor, was applied. The results showed, in the context of TGF-β1 inhibition, that P.g-LPS could not increase the protein expression of Cofilin 2, confirming that it was TGF-β1 that was responsible for Pg-LPS-induced Cofilin 2 upregulation in SK-N-SH APPwt cells (Figures 6C,D). Furthermore, TGF-β1 inhibition could also decrease the phosphorylation of PP2A and Tau at abundant sites (only Ser199 was shown) (Figures 6E,F).

## Discussion

At present, since the cholinergic hypothesis and β-amyloid hypothesis cannot explain the pathogenesis of AD, several anti-AD drugs have failed in phase II and III trials, and currently, effective drugs for the treatment of AD have not yet been identified (Cummings et al., 2019; Sabbagh, 2020). Therefore, determining the pathogenesis of AD from another perspective, which may become the main direction of studies and treatment of AD in the near future, is urgently needed. Periodontitis is a chronic infectious disease of the periodontal tissue, which often leads to the destruction of periodontal support tissue and tooth loss. Statistical studies have shown that the incidence of CP in AD patients is higher than that of non-AD patients of the same age. In addition, CP patients are at higher risk of AD than non-CP patients of the same age, and CP patients’ cognitive ability has declined sharply (de Oliveira Araujo et al., 2021). However, currently, there is a lack of direct evidence reporting the association between the two diseases.

Using the combination of “Alzheimer’s disease and periodontitis “or” Dementia and periodontitis” in pubmed or Scopus databases, a total of 1,088 articles were retrieved. The results show that inflammation, especially neuro-inflammation is the linkage between CP and AD (Dioguardi et al., 2020). *Porphyromonas gingivalis*, the keystone pathogenic bacteria of CP, has been reported to be the chief culprit of Alzheimer’s disease. The inhibition of gingipains, toxic proteases from the bacteria, could reduce the production Aβ_1__–__4__2_, alleviate neuro-inflammation and ultimately rescue neurons of the hippocampus (Dominy et al., 2020). Our previous study also found that the Mini-mental State Examination (MMSE) score of CP patients with 10-year course of disease was significantly lower than that of normal people of the same age. The ratio of serum Aβ_1__–__4__2_/Aβ_1__–__4__0_ in CP patients was significantly lower than that in control group (Rong et al., 2020).

In this study, a new CP animal model was conducted to verify the relationship between CP and AD. The Morris water maze test was used to monitor the spatial learning and memory in the CP mice model. It is well known that APP/PS1 develops cognitive impairment in mice aged 9 months (Wu M. et al., 2017). Therefore, in this study, the CP model was established in mice aged 3 months for a consecutive of 3 months, and the APP/PS1 mice were tested behaviorally at 6 months, when the control mice did not show memory deficits. The results showed that CP could worsen the spatial learning of mice effectively across the 5-day training period. Moreover, probe trials showed the CP mice swam across the hidden platform in the target quadrant less times compared to the control mice in 60 s.

2D-DIGE proteomics study was conducted using the hippocampus from CP mice. A number of different expressed spots were identified, and fifteen of which were detected as significant (Table 1). Some of the identified proteins, such as S100-A9, transthyretin, Cofilin 2, peroxiredoxin 2, and lipocalin-2, have been reported previously in AD and inflammation, which supports our findings and enhances the reliability of this study. These proteins were determined through a series of validation steps. Consistent with the proteomics result, the western blot data showed that the hippocampus of CP mice had significantly higher levels of S100-A9, cofilin 2, peroxiredoxin 2, and lipocalin-2, as compared to the hippocampus of controls (42.3, 85.1, 40.6, and 37.8%, respectively). Nevertheless, no significant difference was found in the level of transthyretin between the two groups, although upward tendency was observed. Cofilin 2 is a well-established protein that has been reported as an AD biomarker in a series of papers. Considering our previous experience, we aimed to determine the expression of Cofilin 2 in the time course of CP pathology. A significant increase of Cofilin 2 level with the development of CP pathology has been demonstrated. The high correlation between the hippocampus level of Cofilin 2 and the time course of CP pathology suggests a specific molecular correlation between CP and AD. This is significantly important because it suggests that Cofilin 2 may serve as a protein link between the two diseases, and the treatment of CP may contribute to attenuate the AD pathology.

As we mentioned earlier, CP is an infectious disease, which is caused by long-term inflammation of the tooth supporting tissue. The pathology of CP is usually accompanied by inflammatory factors, such as IL-1β, IL-6, TNF-α, CRP, and TGF-β1 (Akram et al., 2016). Wu Z. et al. (2017) and Rong et al. (2020) have demonstrated that IL-1β is the key inflammatory mediator that links CP and AD, whereas the results from another study have shown that TNF-α also serves as an important inflammatory factor in the pathogenesis of CP. To determine which inflammatory factor or inflammatory factors are responsible for the up-regulation of Cofilin 2 level in CP, IL-1β, IL-6, TNF-α, CRP, and TGF-β1 are subjected to the supernatant of SK-N-SH APPwt cells, and we found only TGF-β1 treatment could significantly increase the protein level of Cofilin 2. Furthermore, to confirm our result, SB-505124, a specific inhibitor of TGF-β1 receptor, was applied. Western blot analysis showed that TGF-β1 inhibition could effectively reduce the protein level of cofilin 2 triggered by Pg-LPS. Nevertheless future *in vivo* studies are needed to validate this result.

**FIGURE 7:**
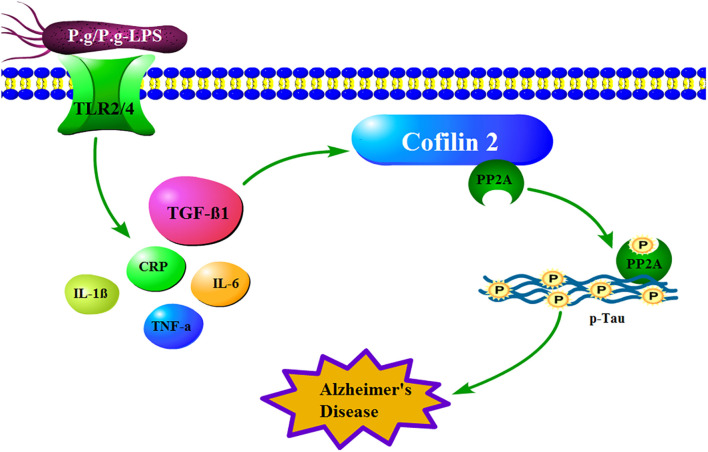
How mechanistically Cofilin 2 acts as an inflammatory linker between CP and AD. In the context of CP, P.g-LPS interacts with Toll like receptor 2/4 and subsequently increases the level of TGF-β1, which induces the protein expression of Cofilin 2 at transcription level. The latter binds and inhibits the activity of PP2A, which leads to the increase of the phosphorylation level of intracellular Tau and eventually induces the pathological changes of AD.

In conclusion, in our behavioral experiment, CP mice showed a more robust memory deficit compared to the control mice. Moreover, in our proteomics study, fifteen differential expressed proteins were identified between the CP and control groups, whereas, in the validation study, S100-A9, transthyretin, Cofilin 2, peroxiredoxin 2, and lipocalin-2 exhibited a significant alteration, which indicated the two diseases was undoubtedly related. Furthermore, the *in vivo* study showed Cofilin 2 might act as a molecular linker between CP and AD, which represents a new target for AD therapy (Figure 7). Our study provides the direct evidence for the association of CP and AD and offers new ideas for the prevention and treatment of AD.

## Data Availability Statement

The original contributions presented in the study are included in the article/Supplementary Material, further inquiries can be directed to the corresponding author/s.

## Ethics Statement

The animal study was reviewed and approved by the Medical Ethics Committee of Shenzhen Baoan Women’s and Children’s Hospital.

## Author Contributions

QZ finished 2D-dige and wrote the article. QF contributed to cell culture and the proteins interaction experiment. XZ provided the idea and direct the experiments. HY was in charge of sample collection. YD contributed to immunofluorescence experiment. WZ and PG contributed to western blot. XR provided the funding of the study and finished the behavior experiment. All authors contributed to the article and approved the submitted version.

## Conflict of Interest

The authors declare that the research was conducted in the absence of any commercial or financial relationships that could be construed as a potential conflict of interest.

## Publisher’s Note

All claims expressed in this article are solely those of the authors and do not necessarily represent those of their affiliated organizations, or those of the publisher, the editors and the reviewers. Any product that may be evaluated in this article, or claim that may be made by its manufacturer, is not guaranteed or endorsed by the publisher.
