# Supplementary material for: Cofilin 2 Acts as an Inflammatory Linker Between Chronic Periodontitis and Alzheimer’s Disease in Amyloid Precursor Protein/Presenilin 1 Mice
Source: Front Mol Neurosci. 2021 Sep 30;14:728184. doi: 10.3389/fnmol.2021.728184 (PMC8514664; doi:10.3389/fnmol.2021.728184)
Supplement: Supplementary file 1 [file Data_Sheet_1.PDF]

Supplementary material

Figure S1. MS spectrum of differentially expressed proteins in the hippocampus of CP mice model as compared with controls

S100-A9

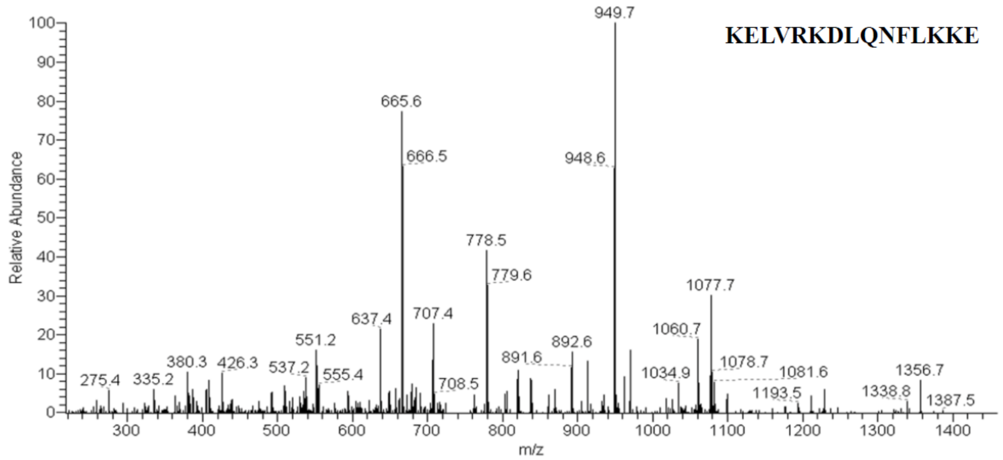

Profilin 2

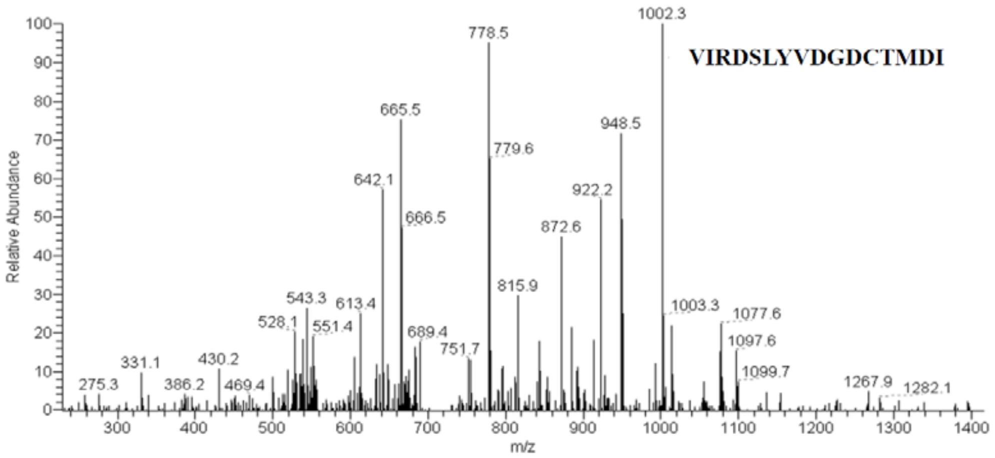

Transthyretin

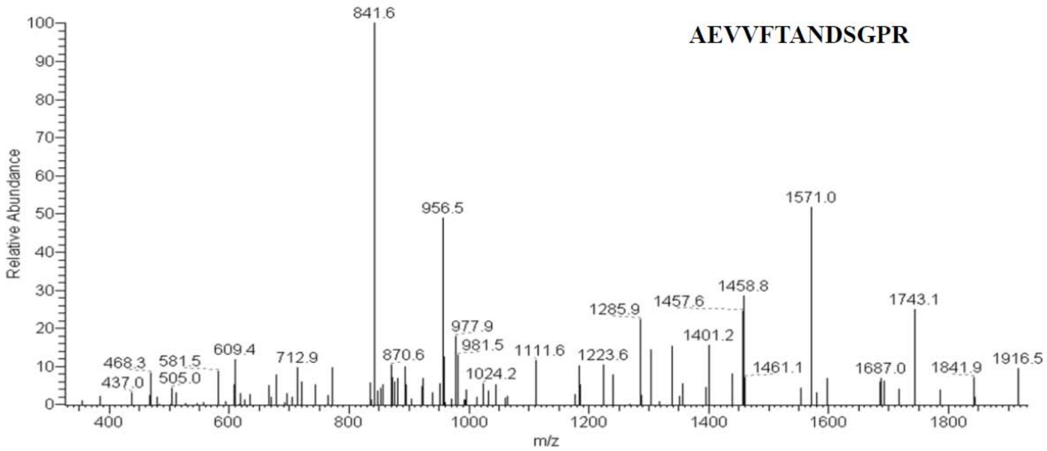

Cofilin 2

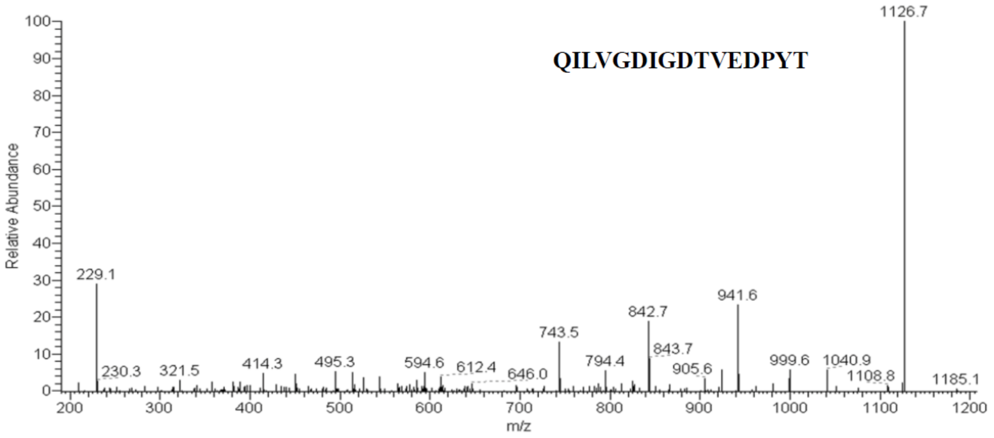

Peroxiredoxin 2

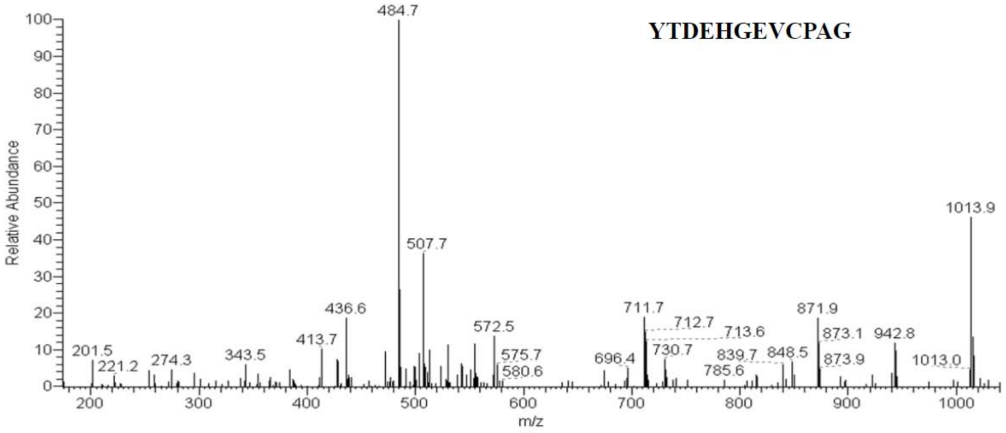

Glutathione peroxidase 4

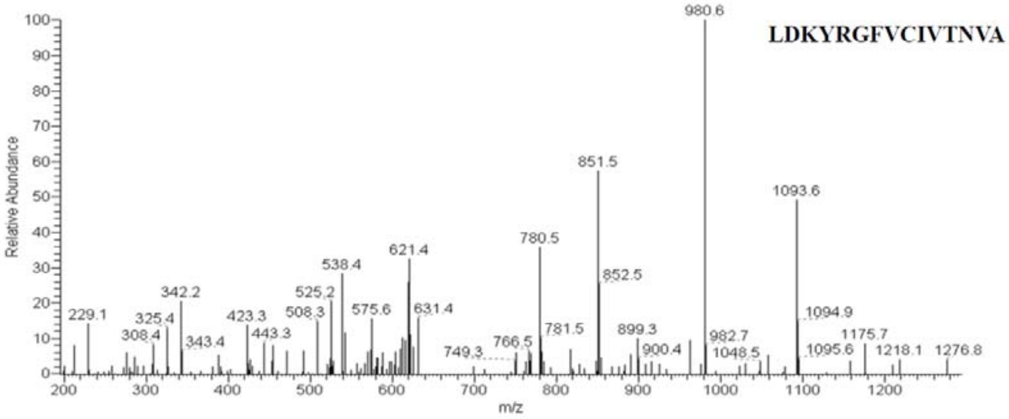

Lipocalin-2

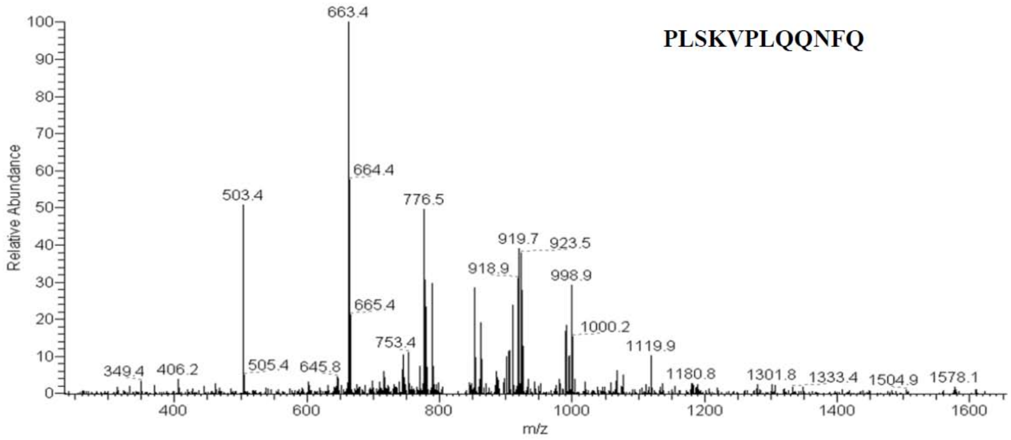

14-3-3 protein sigma

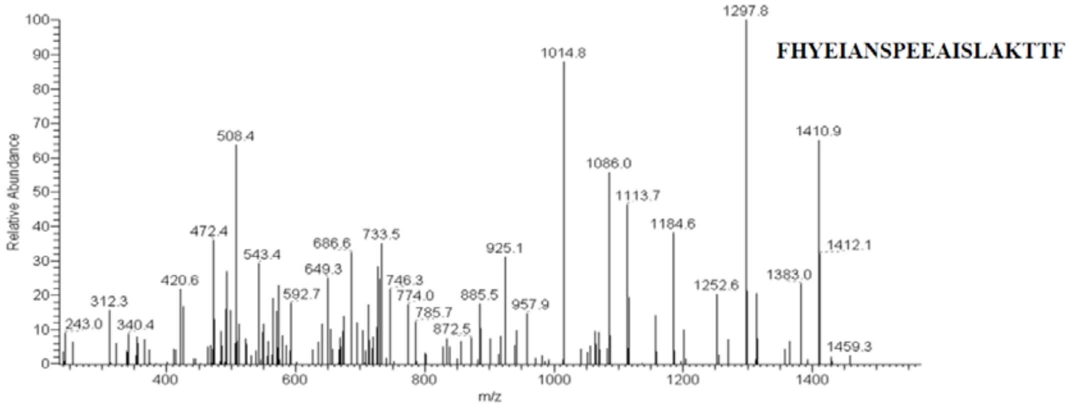

Fibrinogen gamma chain

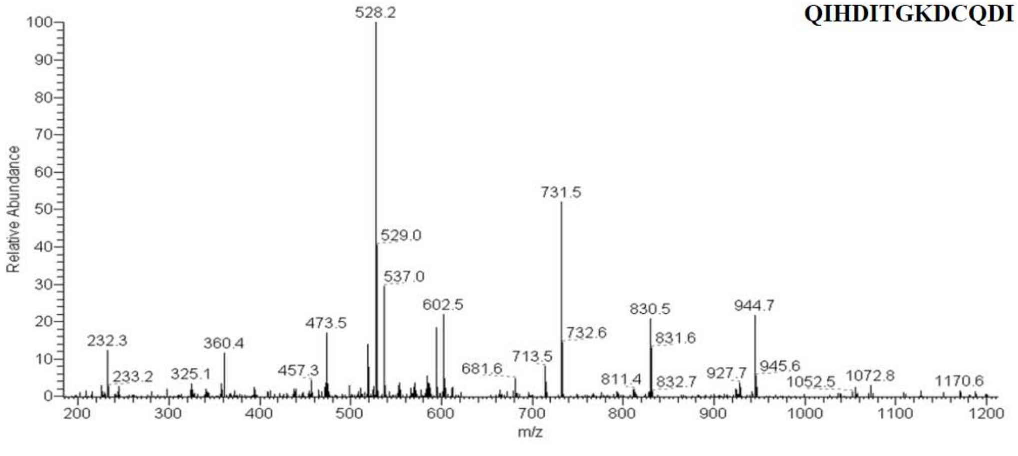

Alpha-tubulin 2

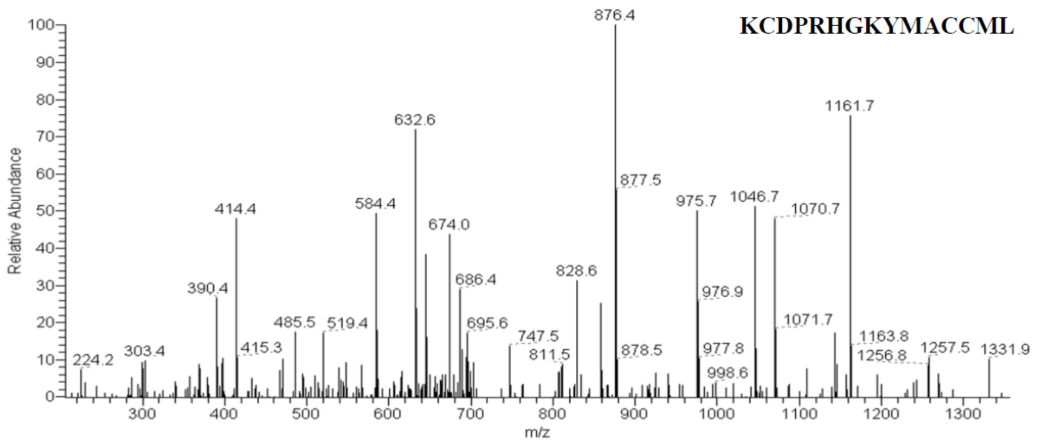

Synapsin-2 isoform IIb

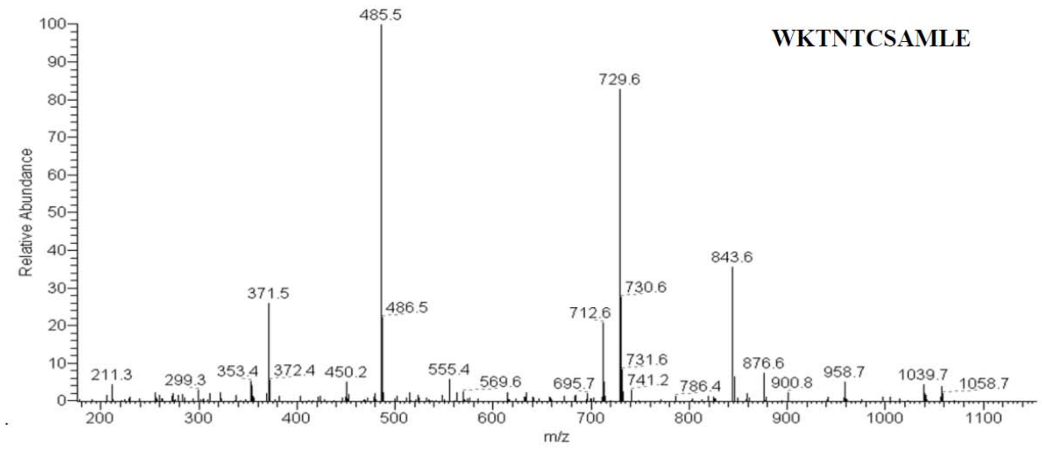

Keratin, type II cytoskeletal

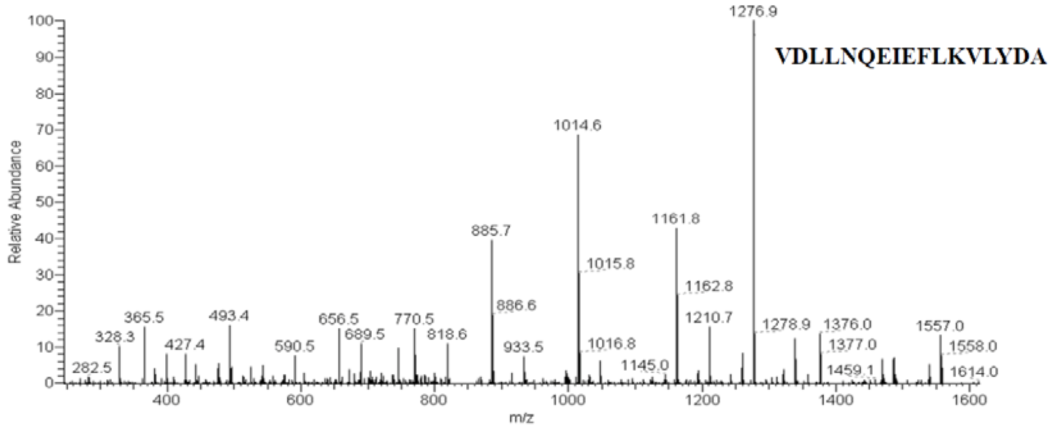

Prothrombin

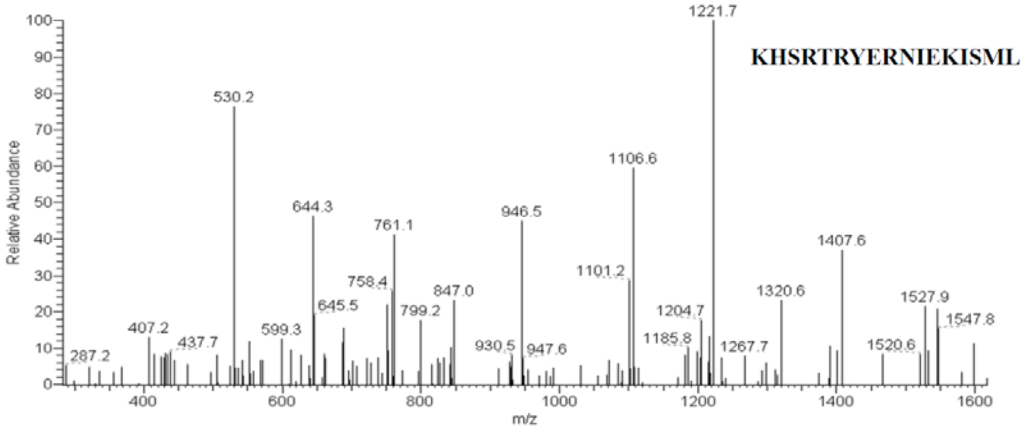

Glycogen phosphorylase

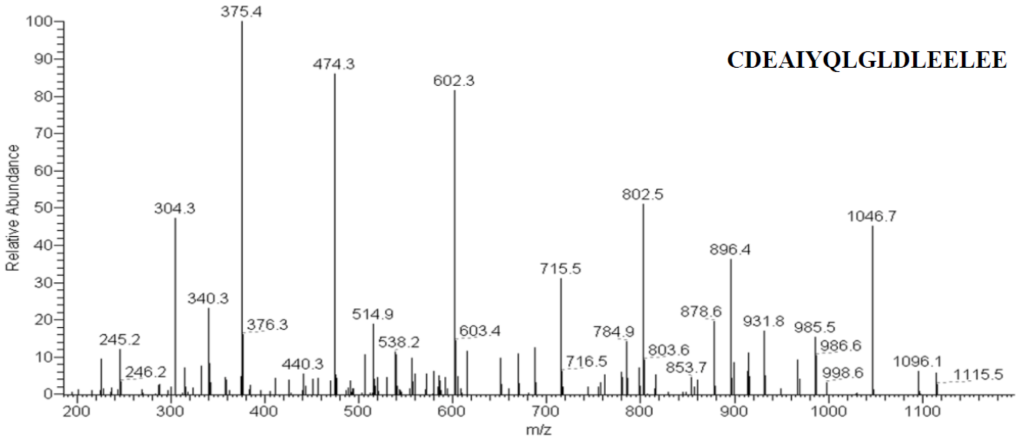

Dynamin-1

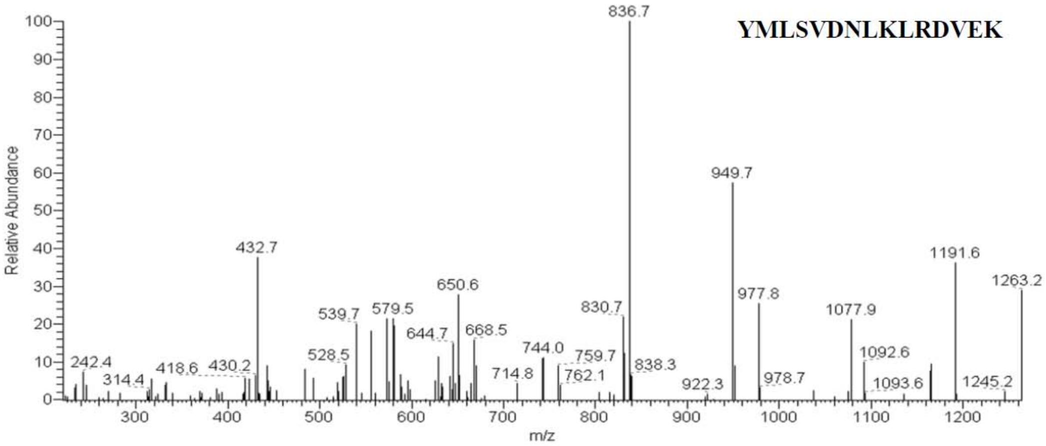

Supplementary material

Cofilin 2 knock-down with adeno-associated virus could partially improve the cognitive impairment of APP / PS1 CP mice

When APP / PS1 mice were 3 months old, the CP model was constructed, and the AAV2-shCofilin 2 was constructed and administered in the lateral ventricle. At the age of 6 months, APP / PS1 mice were tested by water maze. The results showed that the interference efficiency of AAV2-shCofilin 2 was 45.25% compared with the control group. The cognitive impairment of AAV2-shCofilin 2 group was improved at 6 months, but there was no significant difference between the two groups (*p*=0.067). In the exploration experiment, the average crossing time of the control group was 0.8 times in 60s, and the average crossing time of AAV2 shCofilin 2 group was 1.6 times (*p*=0.074). Due to the low interference efficiency of adeno-associated virus, Cofilin 2 gene knockout mice were constructed for later experiments.

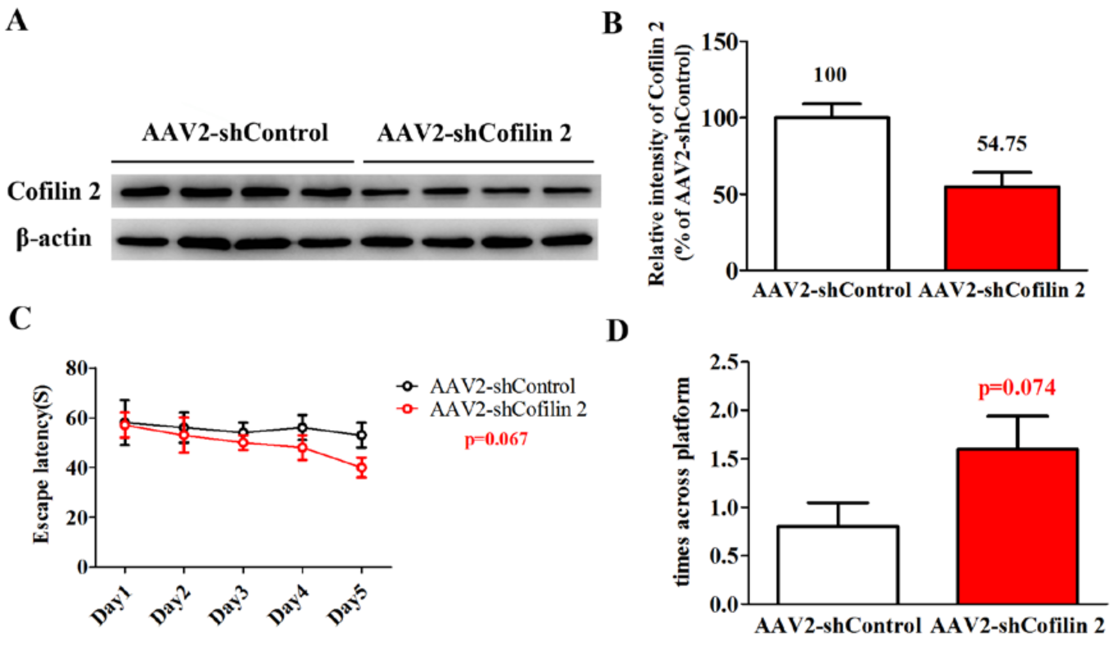

Figure S2. (A. B) Representative panel and quantitative comparison of Western blots of Cofilin 2 in AAV2- shControl group and AAV2-shCofilin 2 group. (C) Latency score represents the time taken to the platform once the mouse was put in the water. (B) Times across the platform represent the frequency for a mouse to swim across the circle where the hidden platform located previously. Data represent mean ± SD. n = 10 mice per group.
